# Results of *ala carte* Posteromedial Soft Tissue Release in Idiopathic Clubfoot

**DOI:** 10.5704/MOJ.2107.013

**Published:** 2021-07

**Authors:** S Barik, L Das, AK Yadav, SS Arora, V Singh

**Affiliations:** Department of Orthopaedics, All India Institute of Medical Sciences Rishikesh, Rishikesh, India

**Keywords:** clubfoot, posteromedial release, scar, Ponseti casting

## Abstract

**Introduction:**

The aim of this study is to assess the outcomes of *ala carte* posteromedial release in children over two years of age who were not responding to the Ponseti method of treatment of idiopathic clubfoot.

**Material and Methods:**

A retrospective observational study from September 2013 to August 2015 was conducted at a tertiary level medical teaching institution. The clubfeet were classified according to the Harold and Walker classification. Radiographic parameters assessed were the talocalcaneal angle (AP, lateral), talus-first metatarsal angle (AP, lateral) and calcaneal-fifth metatarsal angle. The scar and the functional score, according to Laaveg and Ponseti, were evaluated as outcome measures at the final follow-up.

**Results:**

Twenty-four children with a mean age of 43.7 ± 24.7 months were enrolled in the study. There was a total of 36 clubfeet: 21 (65.6%) with a poor functional outcome; 12 (37.4%) with excellent to good scar in both horizontal and vertical components. There was a statistical significance between the pre-operative and post-operative radiological parameters (p<0.05). None of the patients presented with any limitation of activities of daily living despite the poor functional outcome in many of the children. There was no significant association between the qualities of scar (horizontal, vertical) and the functional outcome with age at presentation, pre-operative Harold and Walker classification and pre-operative radiographic angles.

**Conclusion:**

Surgical intervention in terms of *ala carte* posteromedial soft tissue release could not produce a good outcome over four years in CTEV. The threshold for surgery in CTEV should be high, given the poor results.

## Introduction

Congenital talipes equinovarus (CTEV) is one of the most common musculoskeletal deformities. The incidence can be as high as 1.24 per 1000 live births^1^. The Ponseti method has become the first line of treatment for this condition over the last three decades, but it is not without its share of controversies^2^. Results have been uniformly good in up to 90% of cases if the Ponseti treatment is started at an early age^3^. Primary surgical intervention has a very limited role since it leads to a stiff, painful foot over the long term follow-up^4^. However, there are occasions when surgical intervention would be required for a plantigrade pain-free and aligned feet without the need for any footwear modification: the rare clubfeet with deformities not amenable to conservative treatment; the non-compliance with bracing with the persisting clubfoot; and the delayed presentation of patients in developing countries where a conservative treatment alone might not be successful.

The surgical interventions might be in the form of tendon transfers and lengthening, bony procedures or a combination of these. Soft tissue release of the posteromedial structures is performed with midfoot correction followed by correction of the hindfoot deformity^2^. The procedure is performed *ala carte*, unlike those described by Turco or McKay, which are more extensive^7^. There are further modifications described with the release of plantar muscles to correct the cavus and forefoot deformity^8^. The results are mixed, both good and poor, for these extensive surgeries, in the different series^9,10^. The best results of soft tissue releases are obtained in younger children two years of age and below, with outcomes worsening as age increases^11^.

The aim of this study was to determine the outcomes of the *ala carte* posteromedial soft tissue release in children with CTEV two years of age and older who were not responding to the Ponseti method of treatment.

## Materials and Methods

This is a retrospective observational study conducted at a tertiary level medical teaching institution. Data collection was done after informed consent was obtained from parents and approval from the institutional ethical committee. The study period was from September 2012 to August 2015. A minimum follow-up of 48 months (48 – 72 months) was obtained for all the operated clubfeet. The inclusion criteria for this study were children over two years of age, with a failed Ponseti method of casting, and who were treated by the *ala carte* posteromedial soft tissue release. The three criteria for surgical intervention after a Ponseti casting were a clubfoot not improving in appearance; a stiff talonavicular subluxation; and the equinus not amenable to casting. The clubfeet which were syndromic and had earlier surgical correction were excluded.

**Fig. 1:**
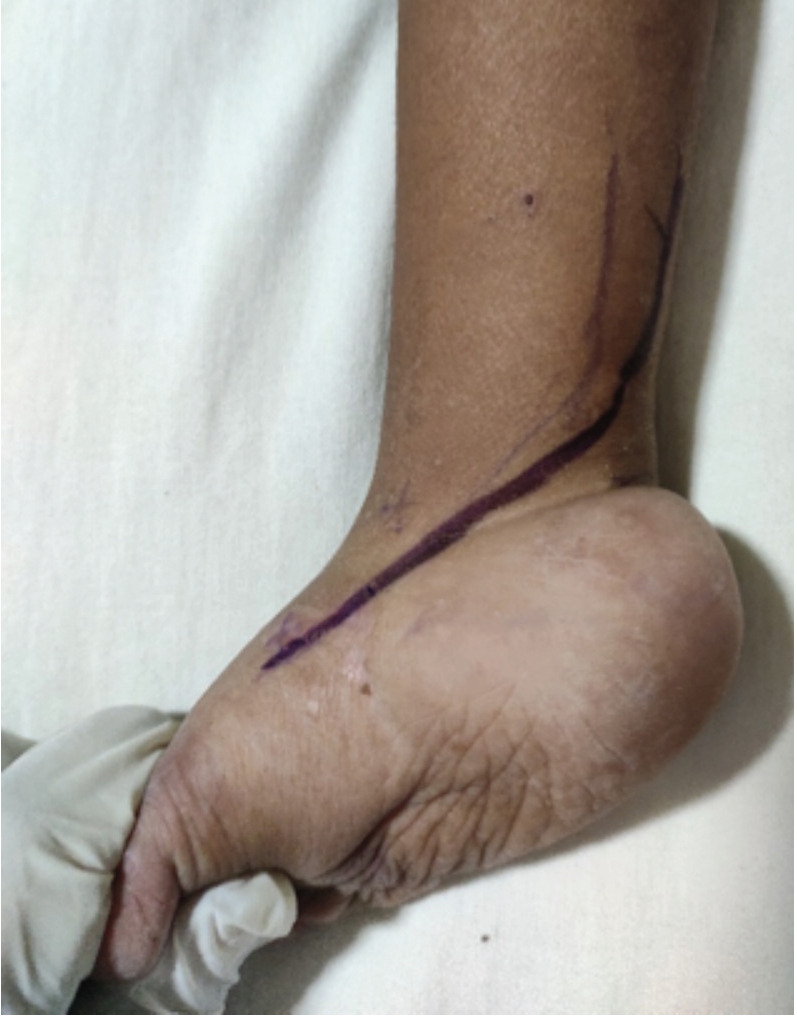
Incision on the foot extending from base of 1st metatarsal to tendoachilles proximally and posteriorly. The scar is divided into two components – horizontal and vertical according to their orientation.

The surgical procedure was carried out under tourniquet control. The plantar and medial release was performed first, followed by the posterior release. The incision curved from the base of the 1st metatarsal anteromedially to the tendoachilles around three cms above the ankle joint posteriorly (Fig. 1). The release of specific structures was decided intra-operatively depending on the tightness of the structures ,preventing the correction of the deformity. After releasing one structure, the following structure to be released was based on the residual deformity.

The order of posterior release included the lengthening of the tendoachilles, the ankle and subtalar capsulotomy and the calcaneofibular ligament. This release corrected the equinus deformity. The sequence of plantar and medial release included the plantar fascia, lengthening of tibialis posterior, toe flexor tendons along with the release of the Master knot of Henry, talonavicular capsular release and the interosseous talocalcaneal ligament. The release of one or more of these structures would reduce the talonavicular joint concentrically and align the 1st metatarsal with the talus. The lengthened tendons are sutured with the foot in a corrected position. An ancillary procedure in the lateral column shortening by the Evans procedure was done in clubfeet with a long lateral column. The correction was maintained by plaster cast in a clubfoot undergoing only soft tissue release, whereas a thick k-wire/pin was used to stabilise the foot undergoing the lateral column shortening. Immobilisation was continued for three weeks with suture removal as an outpatient procedure.

This immobilisation was continued for an additional three weeks for clubfeet that had undergone the lateral column shortening. The implant was then removed at six weeks. Custom made ankle foot orthosis to hold the feet in a corrected position was used during the day. Its usage was continued for a period of 24 months after surgery. Stenbeek foot abduction brace was used at night till the child was four years of age. Exercises in the form of foot abduction, dorsiflexion and squatting were advised. The patients were followed three-monthly in the first year and then six-monthly. Complications and relapse of the operated feet were noted. Repeat casting was done in feet in which the correction achieved was not satisfactory.

**Fig. 2:**
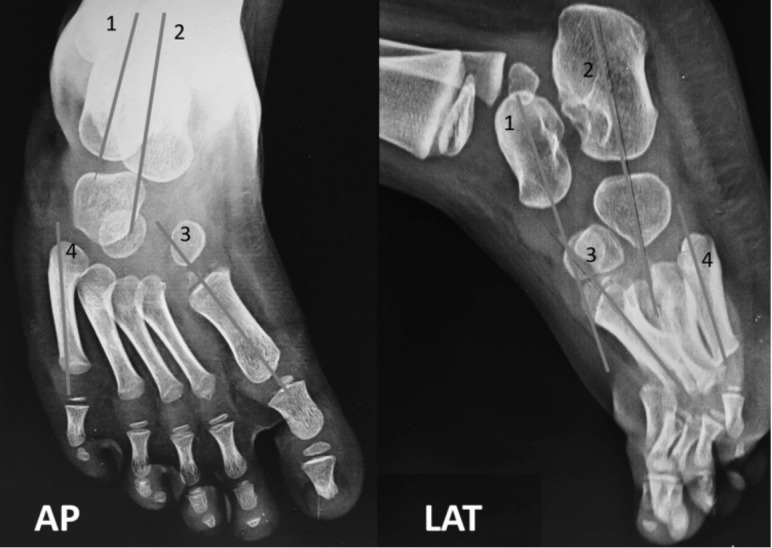
Anteroposterior and lateral image of the foot showing the radiographic parameters used. (1) Axis of talus. (2) Axis of calcaneum. (3) Axis of 1st metatarsal. (4) Axis of 5th metatarsal. Corresponding angles between these axes were measured.

Demographic data of age, sex and laterality of the foot were noted. Pre-operative classification of the CTEV was done according to the Harold and Walker classification^12^. This classification takes into account whether the feet can be corrected beyond neutral (Grade 1) or if the fixed equinus or varus is less (Grade 2) or more (Grade 3) than 20°. Radiographic assessment was made with routine CTEV radiographs, which included the AP view of the foot with ankle, lateral view of the foot with leg and forced dorsiflexion views. Radiographic parameters assessed were the talocalcaneal angle (AP, lateral), the talus-first metatarsal angle (AP, lateral) and the calcaneal-fifth 5th metatarsal angle (Fig. 2). Other parameters assessed at the final follow-up were the condition of the scar and the functional score by Laaveg and Ponseti scoring system (Table I)^2,13^.

**Table I:** Criteria for assessment of the scar at final follow-up

Width of the scar	Length of scar involved	Score
< 2 mm	Full length	5
> 2 mm	< 50 % length	4
	> 50 % length	3
2 - 4 mm	< 50 % length	2
	> 50 % length	1
> 4 mm	Any part of scar	0
**Scar Hypertrophy**	**Length of scar involved**	**Score**
No	Entire length	5
Mild	< 50 %	4
	> 50 %	3
Moderate	< 50 %	2
	> 50 %	1
Severe	Any part of scar	0
**Scar adherence to deeper structures**	**Length of scar involved**	**Score**
	No adherence	5
	< 25 %	4
	25 %-50 %	3
	50 %-75 %	2
	> 75 %	1
	100 %	0

Notes: 13-15; Good: 11,12; Fair: 9,10; Poor: <9

R statistical Software v3.6.0 [R Statistical Corp, Vienna, Austria] was used for data analysis. Descriptive statistics were elaborated in the form of means and standard deviations for continuous variables, and frequencies and percentages for categorical variables. Group comparisons were made using Kruskal - Wallis test, with posthoc pairwise comparisons being made using the Dunn test. Paired t-test/Wilcoxon signed-rank test were used to compare paired variables over time. Statistical significance was set at p < 0.05.

## Results

Twenty four children with a mean age of 43.7 ± 24.7 months were enrolled in the study. Three patients were lost to follow-up. There was a total of 36 club feet, with a final analysis of 32 clubfeet in 21 children (16 males, five females). Eleven children had bilateral clubfeet. All the patients had undergone incomplete Ponseti casting. None of them reported full correction after the casting period, and they were labelled as resistant cases. Ten clubfeet were classified as Grade 1 (mean age 24.8 months), while 13 and 9 were classified as Grade 2 (mean age 40.1 months) and Grade 3 (mean age 70 months), respectively. The severity of deformity increased with increasing age.

At the final follow-up, using the Ponseti Laaveg functional score, poor functional outcome was noted in 21 (65.6%), excellent and good outcomes were noted in 6 (18.7%) (Table II). Residual varus was present in 16, whereas none had any residual equinus. The quality of the scar, both horizontal and vertical components, was poor in most cases. Only 12 (37.4%) had excellent to good scar in both horizontal and vertical components. There was significant difference in the pre-operative and post-operative values of the AP talocalcaneal angle, lateral talocalcaneal angle, AP talus-first metatarsal angle, lateral talus-first metatarsal angle and calcaneal-fifth metatarsal angle (Table III).

**Table II:** Clinical and functional outcome in terms of quality of scar and Ponseti Laaveg score

	Horizontal (n, %)	Vertical (n, %)	Functional score (n, %)
Excellent	2 (6.2%)	2 (6.2%)	2 (6.2%)
Good	10 (31.2%)	10 (31.2%)	4 (12.5%)
Fair	4 (12.5%)	7 (21.9%)	5 (15.6%)
Poor	16 (50.0%)	13 (40.6%)	21 (65.6%)

**Table III:** Radiographic parameters, pre-operative as well as post-operative values

Parameters	Pre-operative (IQR)	Post-operative (IQR)	Wilcoxon test V (p value)
AP talocalcaneal angle	11.56 ± 6.56 (4.00)	17.53 ± 5.62 (6.50)	4.5 (< 0.001)
Lateral talocalcaneal angle	12.53 ± 9.14 (11.50)	22.66 ± 6.65 (8.50)	1.5 (< 0.001)
AP talus 1st metatarsal angle	32.94 ± 11.07 (13.25)	24.66 ± 9.20 (10.50)	361.5 (< 0.001)
Lateral talus 1st metatarsal angle	27.62 ± 11.18 (14.25)	14.94 ± 8.29	401.0 (< 0.001)
Calcaneal 1st metatarsal angle	137.34 ± 13.39 (12.25)	152.75 ± 15.76 (22.50)	55.5 (< 0.001)

IQR – Interquartile range

There was no significant association between the qualities of the scar (horizontal, vertical) and the functional outcome with the age at presentation, the pre-operative Harold and Walker classification and the pre-operative radiographic angles (Table IV, V). Four clubfeet had immediate postoperative complications of wound dehiscence and superficial infection, which were treated conservatively with regular dressings and a plaster holiday of two weeks. A relapse was noted in seven, which included the four mentioned above, with complications managed conservatively by repeat corrective casts by the Ponseti technique. None of the patients presented with any limitation of activities of daily living, despite the poor functional outcome in most of the cases.

**Table IV:** Correlation of the vertical scar quality with age, preoperative classification and preoperative radiographic angles

Parameters	Quality Of Scar (Vertical): Excellent (n = 2)	Quality Of Scar (Vertical): Fair (n = 10)	Quality Of Scar (Vertical): Good (n = 7)	Quality Of Scar (Vertical): Poor (n = 13)	p value	Quality Of Scar (Horizontal): Excellent (n = 2)	Quality Of Scar (Horizontal): Fair (n = 10)	Quality Of Scar (Horizontal): Good (n = 4)	Quality Of Scar (Horizontal): Poor (n = 16)	p value
Age (months)	36.00 ± 0.00	50.40 ± 20.24	29.14 ± 11.19	47.69 ± 31.99	0.2321^1^	24.00 ± 16.97	44.40 ± 23.19	39.00 ± 22.72	47.00 ± 27.39	0.524^1^
H and W Classification					0.639^1^					0.420^1^
Class 1	1 (50.0%)	3 (30.0%)	1 (14.3%)	5 (38.5%)		0 (0.0%)	2 (20.0%)	2 (50.0%)	6 (37.5%)	
Class 2	1 (50.0%)	3 (30.0%)	5 (71.4%)	4 (30.8%)		2 (100.0%)	6 (60.0%)	1 (25.0%)	4 (25.0%)	
Class 3	0 (0.0%)	4 (40.0%)	1 (14.3%)	4 (30.8%)		0 (0.0%)	2 (20.0%)	1 (25.0%)	6 (37.5%)	
Talocalcaneal Angle (AP) (Pre-operative)	7.00 ± 1.41	10.80 ± 5.59	13.29 ± 9.16	11.92 ± 6.30	0.486^1^	8.00 ± 0.00	13.50 ± 8.62	7.25 ± 3.50	11.88 ± 5.71	0.311^1^
Talocalcaneal Angle (Lateral) (Pre-operative)	11.00 ± 4.24	11.50 ± 9.38	13.57 ± 9.47	13.00 ± 10.07	0.949^1^	6.00 ± 2.83	13.80 ± 11.78	12.75 ± 1.50	12.50 ± 9.03	0.637^1^
Talo-1St Metatarsal angle (AP) (Pre-operative)	35.00 ± 4.24	33.50 ± 11.20	32.71 ± 10.81	32.31 ± 12.70	0.996^1^	39.50 ± 2.12	29.90 ± 11.38	36.00 ± 6.48	33.25 ± 12.35	0.537^1^
Talo-1st Metatarsal Angle (Lateral) (Preoperative)	30.00 ± 2.83	29.20 ± 9.87	20.43 ± 12.20	29.92 ± 11.65	0.249^1^	29.50 ± 2.12	23.40 ± 13.04	25.25 ± 7.41	30.62 ± 11.03	0.300^1^
Calcaneal 5th Metatarsal Angle (Lateral) (Pre-operative)	136.00 ± 5.66	135.70 ± 12.75	136.71 ± 16.11	139.15 ± 14.24	0.941^1^	140.50 ± 0.71	135.70 ± 15.66	133.75 ± 9.50	138.88 ± 14.01	0.574^1^

1: Kruskal Wallis Test, 2: Fisher's Exact Test

**Table V:** Correlation of the Ponseti Laaveg functional score with age, pre-operative classification and pre-operative radiographic angles

Parameters	Functional Outcome: Excellent (n = 2)	Functional Outcome: Good (n = 4)	Functional Outcome: Moderate (n = 5)	Functional Outcome: Poor (n = 21)	p value
Age (months)	30.00 ± 8.49	48.00 ± 29.39	28.80 ± 6.57	47.81 ± 26.67	0.378^1^
H and W Classification					0.254^2^
Class 1	2 (100.0%)	0 (0.0%)	2 (40.0%)	6 (28.6%)	
Class 2	0 (0.0%)	2 (50.0%)	3 (60.0%)	8 (38.1%)	
Class 3	0 (0.0%)	2 (50.0%)	0 (0.0%)	7 (33.3%)	
Talocalcaneal Angle (AP)	15.00 ± 12.73	9.25 ± 1.89	15.80 ± 10.26	10.67 ± 5.43	0.844^1^
(Pre-operative)					
Talocalcaneal Angle	24.50 ± 14.85	5.50 ± 1.91	15.80 ± 12.85	11.95 ± 7.59	0.126^1^
(Lateral) (Pre-operative)					
Talo-1st Metatarsal	21.50 ± 14.85	37.75 ± 9.25	28.20 ± 16.45	34.24 ± 9.33	0.463^1^
angle (AP) (Pre-operative)					
Talo-1st Metatarsal	19.00 ± 18.38	33.75 ± 5.62	21.80 ± 18.14	28.67 ± 9.04	0.605^1^
Angle (Lateral) (Pre-operative)					
Calcaneal 5th Metatarsal	146.00 ± 19.80	136.75 ± 11.50	148.80 ± 19.25	133.90 ± 10.56	0.323^1^
Angle (Lateral) (Pre-operative)					

^1^ : Kruskal Wallis Test, ^2^ : Fisher's Exact Test

## Discussion

CTEV is one of the most common congenital musculoskeletal deformities with a male predominance. Up to 50% of cases occur bilaterally^14,15^. This study is also in agreement with the other studies regarding these findings. All the patients in this study had undergone unsuccessful Ponseti casting, which was either discontinued by the parents or failed to attain full correction of the deformity even after the application of many casts. This could be attributed to the poor socio-economic and educational background of the parents in a developing country. The follow-up rate in this study was 87.5% which was comparable to other studies^16,17^.

Poor functional outcome was found in 65.6% of the clubfeet in this study. Although the literature showed good results obtained in CTEV with soft tissue release, this was not the case in our study^18,19^. The patients in studies showing good results were younger compared to the patients in this study. The poor outcome in this study could be attributed to the sparing of the posterolateral structures, the older age at presentation, the superficial infections needing plaster holiday and the hypertrophy of the scar. The nature of the release, both medially and posteriorly, could also be a reason for poor outcome. Dobbs *et al* reported poor long term outcome in patients who had undergone extensive soft tissue release in CTEV with a minimum follow-up of 30 years^4^. He insisted that the early favourable outcomes of extensive soft tissue release would eventually deteriorate over time and that they should be followed up till adulthood to gauge the actual outcome. This poor outcome compared to the club feet treated with Ponseti manipulation was also shown by Ippolito, where he attributed the poor results to the foot and ankle osteoarthrosis that developed over time, the ankle stiffness and the weakness of the gastrocsoleus^20^.

A comparison of the results of this study was made with the Ponseti casting by Laaveg and Ponseti and the extensile soft tissue release by Dobbs *et al*^2,4^. Excellent to good results were obtained in 74% of clubfeet by Laaveg *et al*, and in 33% by Dobbs *et al*. This study showed excellent to good results in 18.6% of the clubfeet. Dobbs *et al* noted radiographic changes of osteoarthrosis in 56% of the surgically corrected clubfeet^4^. He also noted the correlation between the extent of soft tissue release and functional impairment. Ippolito noted radiographic osteoarthrosis in 40% of feet treated with extensile soft tissue surgery^20^. Both the studies had a larger sample size and longer follow-up compared to this study.

The quality of scars was also evaluated in this study which had a horizontal and vertical component starting from the first metatarsal base to the medial border of tendoachilles superiorly (Fig. 1). Excellent to good scars were obtained in 37% of clubfeet, with the remaining being fair to poor at the end of the minimum 48 months follow-up. In contrast, in a follow-up of nine months, 81% of the scars were graded as excellent to good by Joseph, who evaluated the hemi Cincinnati incision^13^. This good outcome of the scars could be attributed to the short follow-up of nine months. With increasing age and growth of the feet, the characteristics of the scar could be expected to change as in our study. The incision evaluated in our study was the Turco incision. It was found that the posterior one-third of the incision was more problematic with issues of scar adherence, scar hypertrophy and widening; and both the horizontal and vertical components of the scar had a negative outcome in more than half of the cases. The scar was also an important limiting factor while evaluating the Laaveg Ponseti score in the patients who had a poor outcome in this study.

The limitations of this study were its retrospective nature, short follow-up and a small cohort. This was the first study, probably, to evaluate the scar outcome in a Turco incision. As the Ponseti method has gained wide acceptance in the treatment of CTEV, planning of randomised controlled trials to evaluate outcomes of surgical procedures in CTEV is not without its ethical and legal issues.

## Conclusion

Surgical intervention in terms of *ala carte* posteromedial soft tissue release could not produce a good outcome over four years in CTEV. None of the patients presented with any limitation of activities of daily living, even in the presence of poor functional outcome in most cases. The threshold for surgeries in CTEV should be high, given the poor results.
